# The Safety and Efficiency of Tirofiban in Acute Ischemic Stroke Patients Treated with Mechanical Thrombectomy: A Multicenter Retrospective Cohort Study

**DOI:** 10.1155/2020/5656173

**Published:** 2020-04-27

**Authors:** Lili Zhao, Yating Jian, Tao Li, Heying Wang, Zhang Lei, Man Sun, Ye Li, Yiheng Zhang, Meijuan Dang, Wang Huqing, Sun Hong, Zhang Ru, Hongxing Zhang, Yi Jia, Luo Guogang, Zhang Guilian

**Affiliations:** ^1^Department of Neurology, The Second Affiliated Hospital of Xi'an Jiaotong University, Xi'an 710004, Shaanxi, China; ^2^Department of Neurology, The First Affliated Hospital of Xi'an Jiaotong University, Xi'an 710000, Shaanxi, China; ^3^Department of Neurology, Xi'an Gaoxin Hospital, Xi'an 710000, Shaanxi, China

## Abstract

**Introduction:**

Limited comparative studies have reported the safety and efficacy of tirofiban in acute ischemic stroke (AIS) patients after mechanical thrombectomy (MT). Additionally, the available studies are inconsistent with each other, which makes application of tirofiban unclear in neuro-intervention. Here, we performed a comparative retrospective study to investigate whether tirofiban combined with MT improves short- and long-term prognosis in AIS patients and whether its use is associated with complications.

**Method:**

Retrospective data were collected for AIS patients admitted between January 2013 and January 2019 at three stroke centers. According to whether tirofiban was used during the operation, patients were divided into tirofiban group and control group. Multivariate and COX regression analyses were performed to determine the association of tirofiban treatment with safety and efficiency in subjects treated with MT.

**Result:**

A total of 174 patients were analyzed, of whom 89 (51.1%) were treated with tirofiban. There were no differences in the incidence of symptomatic intracerebral hemorrhage (10.2% *vs.* 10.6%, *p*=0.918), parenchymal hemorrhage type 2 (18.0% *vs*. 16.5%, *p*=0.793), and reocclusion at 24 h (3.4% *vs*. 10.6%, *p*=0.060) between the tirofiban group and control group. Multivariate regression showed that tirofiban was not associated with intracerebral hemorrhage, early neurological deterioration, neurological improvement at 7 days, functional independence at 3-month and 9-month follow-up, or death at 9-month follow-up (adjusted *p* > 0.05 for all). However, AIS patients treated with MT + tirofiban showed a trend towards acquiring faster functional independence, with a median time to acquire functional independence of 4.0 months compared with 6.5 months in the control group (risk ratio = 1.49, 95% confidence interval 0.98–2.27; long rank *p*=0.066).

**Conclusion:**

Tirofiban may help AIS patients given MT to gain functional independence faster, without increasing the risk of complications.

## 1. Introduction

Mechanical thrombectomy (MT) has become the first-line treatment for acute ischemic stroke (AIS) within 6 h of onset and is recommended by health guidelines [1]. However, after successful recanalization, approximately 2%–20% of patients undergo reocclusion, which leads to an unfavorable prognosis [2]. It is generally accepted that the intravascular operative procedure causes endothelial damage [3], plaque disruption, and subsequent platelet activation, resulting in early reocclusion [2, 4]. Tirofiban is a highly selective, reversible, low-molecular-weight nonpeptide platelet GP IIb/IIIa receptor antagonist. Tirofiban prevents fibrinogen binding to platelets and subsequent platelet aggregation by blocking the final step of platelet activation [5]. In theory, tirofiban may be more effective than other existing antiplatelet agents.

Tirofiban has been recommended by health guidelines as routine antiplatelet therapy for percutaneous coronary angioplasty in acute myocardial infarction [6]. However, as an off-label usage, the clinical evidence of tirofiban in AIS is limited. In 2011, the Safety of Tirofiban in Acute Ischemic Stroke trial initially demonstrated the relative benefits of intravenous (IV) tirofiban in AIS patients. A number of following studies have reported effects of tirofiban in IV thrombolysis [7, 8], emergency permanent stenting [9], angioplasty, and endovascular thrombectomy [9–16]. However, many of these were observational studies without controls and with a limited number of cases [7, 9, 11, 14, 16], single-center studies within relative short follow-up interval (approximately 90 days), or had a varied tirofiban dosing regimen [10, 12, 13, 15]. The findings also varied in terms of symptomatic intracerebral hemorrhage (sICH), functional outcome, and mortality [10, 12, 13, 15, 17] or did not find that tirofiban promoted functional independence [12, 18]. Thus, we performed a multiple center study of follow-up postoperative functional outcomes at 3 and 9 months' recovery to examine the relationship between tirofiban combined with MT and intracerebral hemorrhage (ICH), short- and long-term prognosis, and death in AIS patients.

## 2. Methods

### 2.1. Population and Research Design

This is a retrospective analysis of the cohorts of the 3 comprehensive stroke centers between January 2013 and January 2019. We selected AIS patients who underwent MT from the Department of Neurology, The Second Affiliated Hospital of Xi'an Jiaotong University, The First Affiliated Hospital of Xi'an Jiaotong University and Xi'an Gaoxin Hospital. The patients who met the recommended guideline criteria for IV thrombolysis received IV recombinant human tissue plasminogen activator (rt-PA) prior to MT. Without waiting for alteplase effect, digital subtraction angiography (DSA) was performed as soon as possible. If the clinical symptoms did not improve or there was large vessel occlusion (LVO), MT were performed immediately. For those patients with >6 hours after stroke onset, MT were performed based on their perfusion mismatch between arterial spin labeling (ASL) and diffusion-weighted imaging (DWI), cerebral blood flow (CBF) and cerebral blood volume (CBV), and collateral flow on DSA (≥level 2, according to American Society of Interventional and Therapeutic Neuroradiology/Society of Interventional Radiology (ASITN/SIR) [19]) or Alberta Stroke Program Early CT Score/post-circulation Alberta Stroke Program Early CT Score (ASPECTS/pc-ASPECTS) (≥6 scores) on noncontrast CT or DWI. The inclusion criteria were as follows: (1) symptoms ≤ 8 h and (2) age ≥ 18 years. Exclusion criteria were as follows: (1) symptoms ＞ 8 h, (2) age <18 years, (3) collateral flow on DSA <2 level or ASPCTS/pc-ASPECTS <6 scores, (4) LVO in bilateral ICA or anterior and posterior circulation simultaneously, (5) coexisted severe systematic diseases or dyscrasia, but the families required for MT, (6) with incomplete data, and (7) lost to follow-up. Patients were divided into two groups according to whether tirofiban was used during the operation (tirofiban group and control group).

### 2.2. Correlative Operational Details

All surgeons had >5 years of experience in neuro-intervention and could proficiently perform MT and extracranial/intracranial stents. Local or general anesthesia was chosen according to the level of patient cooperation and the medical condition. IV heparin to maintain the activated clotting time in the range of 200–300 s during the procedure was mandatory, except for subjects treated with IV alteplase. The type and size of other necessary devices (such as guide wires and balloon catheters) and the intervention strategy were left to the surgeons' discretion.

### 2.3. Situations/Parameters Influencing the Use of Tirofiban

Tirofiban was administered at the discretion of the surgeon, based on the following criteria: (1) residual stenosis ≥70% in the occlusion site after thrombectomy with forward blood flow not maintained by modified treatment in cerebral infarction (mTICI) ≥2b over 10 min, (2) rescue treatment with stenting or balloon angioplasty, (3) potential endothelial damage during the procedure (such as thrombectomy attempted three times), (4) distal embolization during the stent retrieval procedure, (5) reocclusion after the first reperfusion, and (6) failed thrombectomy (no anterior blood flow after three attempts).

#### 2.3.1. Tirofiban Use

Tirofiban was administered intra-arterially with a bolus of 5 *μ*g/kg (dose range of 0.25–0.5 mg; Lunan Pharmaceutical Co. Ltd., Shandong, China; standard: 12.5 mg of tirofiban diluted with 250 mL of normal saline), followed by continuous infusion of 0.1 *μ*g/kg/min for at least 24 h (but not more than 36 h) when no obvious ICH was found in follow-up computed tomography (CT) scan (immediately, and 12 h and 24 h after the procedure; reduced by 50% if intracranial high density was found on CT scan immediately after surgery and failed to exclude ICH, or if minor bleeding existed in other regions such as the mouth and urethra). Additionally, some patients were only treated with intra-arterial injection (dose range of 0.15–0.5 mg) or continuous IV infusion (0.1 *μ*g/kg/min for 24 h). If CT showed significant ICH or severe systemic bleeding, infusion was terminated.

### 2.4. Supernumerary Antiplatelet Management

Dual antiplatelet (aspirin 100 mg and/or clopidogrel 75 mg) and IV tirofiban overlapped for 4 h, and then IV tirofiban was stopped. If tirofiban was not used or just used intra-arterially, the preoperative antiplatelet regimen was determined by the surgeon based on whether the patient would receive IV thrombolysis and on previous antiplatelet treatment.

### 2.5. Other Treatments

Patients with pathogeny of cardioembolism or with deep venous thrombosis during hospitalization were treated with anticoagulants depending on their condition. General medical treatments (dehydration, sedative, and neuroprotective drugs) depended on the doctors' discretion.

### 2.6. Data Collection

We obtained the following patient information from the database: baseline characteristics (age, sex, previous transient ischemic attack/stroke, and vascular risk factors), National Institutes of Health Stroke Scale (NIHSS), ASPECTS/pc-ASPECTS, etiology according to the Trial of ORG 10172 in Acute Stroke Treatment (TOAST), operational details (pre rt-PA, anesthesia, location of stroke, and time from onset to groin puncture (OTP)), level of ASITN/SIR, balloon angioplasty, permanent stenting, number of passes, mTICI, emergency laboratory tests (glucose, platelet count, and coagulation), median blood pressure within 24 h, imaging data (CT, DWI, and DSA), treatment in hospital (perioperative antiplatelet, anticoagulation, sedative, and dehydration management), ICH within 7 days, parenchymal hemorrhage type 2 (PH2), sICH, 3-month and 9-month (if available) functional outcomes (modified Rankin score (mRS)), and death at 9 months. The NIHSS score, ASPECTS/pc-ASPECTS score, TOAST, and imaging were performed independently by two researchers. When there was a large discrepancy, the final result was decided jointly by the two researchers.

#### 2.6.1. Study Follow-Up

Patients were followed up by telephone and recalled their neurological function recovery at 3 and 9 months after surgery. We defined mRS ≤2 as the follow-up endpoint. Subjects who died or did not reach the endpoint were censored, and the survival time was calculated monthly.

### 2.7. Outcomes

The outcomes were divided into safety and efficacy outcomes. The main safety outcomes were (1) ICH within 7 d (European Cooperative Acute Stroke Study III (ECASS-III) [20]) and (2) 9-month death. Secondary safety outcomes were (1) PH2 (ECASS-II [21], blood clots in >30% of the infarcted area with substantial space-occupying effect, or with any hemorrhage outside the infarct area including intraventricular hemorrhage and subarachnoid hemorrhage) and (2) sICH within 7 d (ECASS-III; any intracranial hemorrhage associated with worsening clinical symptoms, NIHSS increased by ≥4 points, and no other explanation for neurological deterioration).

The primary efficacy outcomes were (1) 3-month mRS (0–2 points for functional independence) and (2) 9-month mRS. Secondary outcomes were (1) early neurological deterioration (END); NIHSS score increased by ≥2 points within 24 h of onset [22]; (2) neurological improvement at day 7 (defined as a ≥4-point decrease on NIHSS after treatment compared with baseline); and (3) reocclusion within 24 h (reocclusion in this study was defined as NIHSS worsening by ≥2 points after improvement of ≥2 points of the initial NIHSS, excluding intracranial hemorrhage transformation) [7].

### 2.8. Statistical Analysis

Baseline characteristics were expressed as mean ± standard deviation or median with interquartile range (IQR) for continuous variables and as numbers with percentages or ratio for categorical variables. Comparisons between the two groups were performed with the Mann–Whitney *U*-test, *χ*^2^ test, or Fisher exact test, as appropriate. The associations of tirofiban use with categorical outcomes were evaluated using a multivariate logistic regression model. Kaplan–Meier survival analysis was performed to test differences between the two groups (log-rank test), while COX regression analysis was used to assess the relationship between tirofiban use and functional independence. Baseline characteristics showed a univariate relationship with outcome (*p* < 0.1), or clinically relevant variables were included as covariates in the model (entry). Statistical analysis was performed with statistical software (SPSS v19.0; IBM Inc., Armonk, NY, USA; significance level: *p* < 0.05, 2 sided).

## 3. Results

### 3.1. Baseline Characteristics

**Figure 1:**
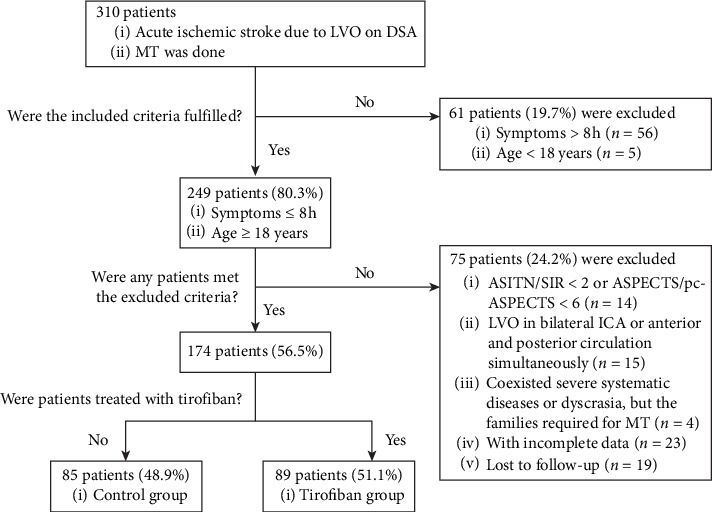
Flow chart of patient inclusion steps. LVO, large vessel occlusion; DSA, digital subtraction angiography; MT, mechanical thrombectomy; ASITN/SIR, American Society of Interventional and Therapeutic Neuroradiology/Society of Interventional Radiology; ASPECTS/pc-ASPECTS, Alberta Stroke Program Early CT Score/post-circulation Alberta Stroke Program Early CT Score.

There were a total of 310 AIS patients due to LVO and MT in three stroke centers from January 2013 to January 2019. One hundred and thirty-six patients were excluded with symptoms ＞8 h, age <18 years, improper collateral circulation, improper ASPECTS/pc-ASPECTS, and incomplete data. Finally, one hundred and seventy-four (56.5%) patients were included in our study. All enrolled patients had follow-up information. The patient flowchart is shown in Figure 1.

Of the 174 patients, 89 (51.1%) are in the tirofiban group, while 85 (48.9%) are in the control group. Tables 1 and 2 summarize the patient' demographic, clinical characteristics, operational details, and perioperative managements in two groups. There were a higher proportion of male sex (66.3% *vs.* 50.6%), smoking (28.1% *vs.* 14.1%), hypertension (56.2% *vs.* 41.2%), and large artery atherosclerosis (76.4% *vs.* 48.2%) in the tirofiban group than that in the control group (*p* < 0.05, each) while the percentage of atrial fibrillation (29.2% *vs.* 60.0%), cardioembolism (23.6% *vs.* 51.8%), and premorbid anticoagulants (4.5% *vs.* 12.9%) were lower in the tirofiban group than that in the control group (*p* < 0.05, respectively). In the tirofiban group, there were a longer OTP time (345 *vs.* 258.5 min), a higher percentage of balloon angioplasty (30.3% *vs.* 7.1%), permanent stenting (23.6% *vs.* 8.2%), operative antiplatelet (84.3% *vs.* 57.6%), a more mTICI ≥2b (76.4% *vs.* 56.5%), sedation (32.6% *vs.* 16.5%), and a lower rate of anticoagulation (19.1% *vs.* 44.7%) than that in the control group (*p* < 0.05, each). The other factors showed no differences in two groups (*p* > 0.05, each).

### 3.2. Efficiency and Safety Outcomes

Table 3 shows that there was a trend for a lower incidence of reocclusion at 24 h in the tirofiban group (3.4%) compared with the control group (10.6%; *p*=0.06). Nineteen subjects (21.3%) treated with tirofiban and 23 subjects (27.1%) not given tirofiban experienced END (*p*=0.379). There were no differences in the rate of neurological improvement at 7 d between the tirofiban group and the control group (48.8% *vs.* 41.7%, *p*=0.381). Further, 51 patients (29.3%) had functional independence at 3-month follow-up, and the percentage of functional independence was significantly higher in the tirofiban group patients compared with the control group (36.0% *vs.* 22.4%, *p*=0.049). At 9-month follow-up, 53 patients (59.6%) in the tirofiban group and 40 patients (47.1%) in the control group acquired functional independence (*p*=0.099).

A total of 68 patients (39.1%) had ICH, and in-hospital ICH occurred more frequently in the control group than in the tirofiban group (45.9% *vs.* 32.6%, *p*=0.072). Of 174 patients, 30 were PH2 (17.2%) and 12 were sICH (10.3%). There were no differences in the rates of PH2 and sICH between the tirofiban group and the control group (18.0% *vs.* 16.5% and 10.2% *vs*. 10.6%, *p*=0.793 and 0.918, each). At 9-month recovery, a total of 50 patients (28.7%) had died. The tirofiban group had a trend for a lower rate of death than that in the control group (23.6% *vs.* 34.1%, *p*=0.125).

### 3.3. Multivariate Regression Analysis

Variables that were identified as clinically relevant or showed *p* < 0.1 on univariate analysis were included in the final multivariate regression analysis model of every outcome. The results of multivariate regression analysis about tirofiban and functional outcomes are shown in Table 4. Tirofiban use was not associated with END, neurological improvement at 7 d, functional independence at 3-month and 9-month follow-up, ICH, and death at 9-month follow-up (adjusted *p* > 0.05 for each).

### 3.4. COX Regression Analysis

**Figure 2:**
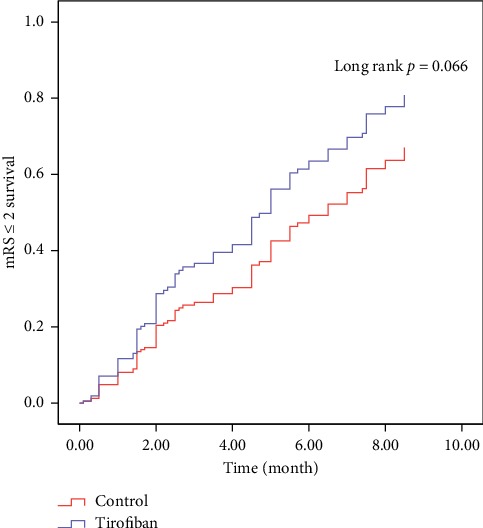
Survive curve of tirofiban treated *vs*. control patients given MT.

There was a trend for a slightly shorter median time to acquire functional independence in the tirofiban group (4.0 months, 95% confidence interval (CI): 2.88–5.12) compared with the control group (6.5 months, 95% CI 5.40–7.60; log-rank *p*=0.127; Table 5). After adjusting for atrial fibrillation and NIHSS on admission, AIS patients treated with MT + tirofiban showed a trend for acquiring faster functional independence (risk ratio = 1.49, 95% CI 0.98–2.27, long rank *p*=0.066; Figure 2 and Table 6).

## 4. Discussion

In this comparative study, we found that the application of tirofiban during MT may be safe and potentially help to improve prognosis in patients treated with MT for AIS. Tirofiban did not significantly increase the risk of bleeding complications or 9-month death. Further, tirofiban was not associated with short- and long-term prognosis. However, tirofiban may allow AIS patients given MT to gain functional independence faster.

Our findings were consistent with several previous studies reporting that endovascular treatment alone was not superior to tirofiban combined with EVT in terms of safety [10, 12, 17, 23]. A recent meta-analysis also demonstrated that tirofiban did not increase the risk of ICH, sICH, or mortality at 3 months [24]. By contrast, a prospective cohort study by Wu et al. [13] concluded that additional tirofiban was associated with increased risk of ICH, sICH, and fatal ICH. The differences between our study and that of Wu et al. may relate to their significantly higher percentage of subjects with atrial fibrillation (total of 56.7%, with 46.7% in the tirofiban group). Previous studies demonstrated a high risk of hemorrhagic transformation in patients with cardiogenic stroke or stroke with atrial fibrillation [25, 26]. To a certain extent, this may explain the favorable safety outcomes observed in the present study. In addition, our multivariate regression showed that anterior circulation stroke (ACS) was an independent risk factor of ICH and 9-month mortality. According to previous studies, ACS patients were more likely to experience ICH [27], sICH, and mortality at 3-month follow-up after receiving IV thrombolysis [28].

The efficacy of tirofiban on prognosis in AIS patients undergoing MT remains controversial [15, 17]. In the present study, there was no benefit of tirofiban on END and neurological improvement at 7 d recovery. Further, our follow-up data show that tirofiban administration was not associated with neurological improvement at 3-month and 9-month recovery, although the tirofiban group showed a trend towards a higher rate of function independence (36.0%, *p*=0.049). Patients in our study also seemed to exhibit a shorter time to achieve function independence after treatment with tirofiban. These findings are very similar to the results of Zhao et al. who reported that tirofiban had no influence on 3-month functional independence, although it did improve long-term (average length of follow-up was 12 months (IQR 3–27)) neurological function improvement. Other studies reporting no benefits of tirofiban were usually performed at 3-month follow-up [12, 18]. Considering our relative short follow-up period of 9 months, more prolonged observation times may be required to determine the effects of tirofiban in patients treated with MT.

To our best knowledge, this is the first study using COX regression to assess the association of tirofiban with function independence time in AIS patients receiving MT. Further, we provide clinical evidence for the use of tirofiban in these cases. However, there are some limitations of our study. First, this was a retrospective cohort study and is therefore affected by selection bias. Second, the dose and usage of tirofiban in this study were inconsistent. Further studies are required to determine the optimal tirofiban treatment protocol in AIS patients treated with MT. Third, in our study, the use of tirofiban was at the discretion of the treating interventionist according to the characteristics of the lesions and procedure situation, which may have caused bias. Finally, because of the limited number of cases with PH2, sICH, and reocclusion at 24 h, multivariate analysis of these outcomes was not performed, and a much larger study is required.

## 5. Conclusions

Our findings show that tirofiban was not associated with higher ICH, PH2, sICH, or short-term and long-term neurological functional changes in AIS patients treated with MT. However, AIS patients treated with MT combined with tirofiban showed a trend for acquiring faster functional independence.

## Figures and Tables

**Table 1:** Demographic and clinical characteristics of the subjects.

Characteristic	Tirofiban group *N* = 89	Control group *N* = 85	*p* value
Sex (male)	59 (66.3)	43 (50.6)	0.036
Age, median (IQR), *y*	68 (56.5–75.0)	68 (54.5–77.0)	0.640
Smoking	25 (28.1)	12 (14.1)	0.024
Drinking	17 (19.1)	9 (10.6)	0.115
Hypertension	50 (56.2)	35 (41.2)	0.048
T2DM	24 (27.0)	17 (20.0)	0.279
Coronary atherosclerotic disease	27 (30.3)	26 (30.6)	0.971
Atrial fibrillation	26 (29.2)	51 (60.0)	0.000
Previous TIA/stroke	17 (19.1)	11 (12.9)	0.269
NIHSS on admission, median (IQR), point	15 (12.0–21.0)	16 (13.0–20.0)	0.452
ASPECTS/pc-ASPECT, median (IQR), point	10 (9.0–10.0)	10 (8.3–10.0)	0.456
Glucose, median (IQR), mmol/l	7.2 (6.2–9.4)	7.2 (5.9–9.0)	0.457
PLT, median (IQR), ×10^9^/L	192 (147.2–236.7)	177 (144.0–213.3)	0.142
INR, median (IQR), *s*	1.03 (0.95–1.11)	1.05 (0.99–1.14)	0.064

*TOAST*			
Large artery atherosclerosis	68 (76.4)	41 (48.2)	0.000
Cardioembolism	21 (23.6)	44 (51.8)	0.000

*Location of stroke*			
Anterior circulation stroke	69 (77.5)	67(78.8)	0.836
Posterior circulation stroke	20 (22.5)	18(21.2)	0.836
Median SBP in 24 h, median (IQR), mmHg	130 (118.0–145.0)	130 (121.3–140.0)	0.926
Premorbid antiplatelets	17 (19.1)	11 (12.9)	0.269
Premorbid anticoagulants	4 (4.5)	11 (12.9)	0.047

Data are number, number (%), median (IQR), or mean (SD).

**Table 2:** Operational details and operative period managements.

Characteristic	Tirofiban group *N* = 89	Control group *N* = 85	*p* value
Pre rt-PA	27 (30.3)	22 (25.9)	0.514
OTP time, median (IQR), min	345 (244.0–419.5)	258.5 (194.0–322.7)	0.000
Local anesthesia	63 (70.8)	70 (82.4)	0.072
ASITN/SIR (1-2)	73 (82.0)	66 (77.6)	0.472
Number of passes, median (IQR)	2 (1–3)	2 (1–2)	0.738
Balloon angioplasty	27 (30.3)	6 (7.1)	0.000
Permanent stenting	21 (23.6)	7 (8.2)	0.006
mTICI ≥ 2b	68 (76.4)	48 (56.5)	0.005

*Operative period antiplatelet*			
None	14 (15.7)	36 (42.4)	0.000
Aspirin/clopidogrel	22 (24.7)	27 (31.8)	
Aspirin + clopidogrel	53 (59.6)	22 (25.9)	

*Postoperative treatment*			
Anticoagulation	17 (19.1)	38 (44.7)	0.000
Sedation	29 (32.6)	14 (16.5)	0.014
Dehydration	64 (71.9)	55 (64.7)	0.307

Data are number, number (%), median (IQR), or mean (SD). OTP time indicated time from onset to groin puncture.

**Table 3:** Efficacy and safety outcomes.

Outcome	Tirofiban group	Control group	*p* value
*Efficiency outcomes*			
Reocclusion at 24 h	3 (3.4)	9 (10.6)	0.060
END	19 (21.3)	23 (27.1)	0.379
Neurological improvement at 7 d	39 (48.8)	30 (41.7)	0.381
mRS ≤ 2 at 3^th^ month	32 (36.0)	19 (22.4)	0.049
mRS ≤ 2 at 9^th^ month	53 (59.6)	40 (47.1)	0.099

*Safety outcomes*			
ICH	29 (32.6)	39 (45.9)	0.072
PH2	16 (18.0)	14 (16.5)	0.793
SICH	9 (10.2)	9 (10.6)	0.918
Death at 9^th^ month	21 (23.6)	29 (34.1)	0.125

Data are number, number (%).

**Table 4:** Multivariate regression analysis to determine the influence of tirofiban on safety and efficacy outcomes.

Outcome	aOR	95% CI	*p* value
*Efficiency outcomes*			
END^a^	1.22	0.54–2.72	0.63
Neurological improvement at 7 d^b^	1.02	0.49–2.12	0.951
mRS ≤ 2 at 3^th^ month^c^	1.54	0.69–3.45	0.296
mRS ≤ 2at 9^th^ month^d^	1.22	0.61–2.46	0.577

*Safety outcomes*			
ICH^e^	0.57	0.28–1.16	0.122
Death at 9^th^ month^f^	0.840	0.39–1.79	0.652

AOR: adjusted OR. ^a^Adjusted operative period antiplatelet, TICI. ^b^Adjusted operative period antiplatelet, median SBP in 24 h,. ^c^Adjusted dehydration, NIHSS on admission, operative period antiplatelet, sedative. ^d^Adjusted NIHSS on admission, operative period antiplatelet. ^e^Adjusted anterior circulation stroke, dehydration, operative period antiplatelet. ^f^Adjusted dehydration, anterior circulation stroke, and operative period antiplatelet.

**Table 5:** Median mRS ≤ 2 survival time.

mRS ≤ 2	Median (months)	95% CI		*p* value
		Lower	Upper	
Tirofiban group	4.0	2.88	5.12	0.127
Control group	6.5	5.40	7.60	

**Table 6:** Cox regression analysis to evaluate the impact of tirofiban on time to acquire mRS ≤ 2.

Outcome	RR	95% CI	*p* value
mRS ≤ 2 *k*	1.49	0.98–2.27	0.066

*k*: adjusted atrial fibrillation, NIHSS on admission.

## Data Availability

The data used to support the findings of this study are available from the corresponding author upon request.
